# In-Vitro Application of a Qatari *Burkholderia cepacia* strain (QBC03) in the Biocontrol of Mycotoxigenic Fungi and in the Reduction of Ochratoxin A biosynthesis by *Aspergillus carbonarius*

**DOI:** 10.3390/toxins11120700

**Published:** 2019-12-02

**Authors:** Randa Zeidan, Zahoor Ul-Hassan, Roda Al-Thani, Quirico Migheli, Samir Jaoua

**Affiliations:** 1 Department of Biological & Environmental Sciences, College of Arts & Sciences, Qatar University, Doha P.O. Box 2713, Qatar; rz1604991@student.qu.edu.qa (R.Z.); zahoor@qu.edu.qa (Z.U.-H.); ralthani@qu.edu.qa (R.A.-T.); 2 Dipartimento di Agraria, Università degli Studi di Sassari, 07100 Sassari, Italy; qmigheli@uniss.it

**Keywords:** Ochratoxin A, biological control, Qatari microflora, *Burkholderia cepacia*, thermostability

## Abstract

Mycotoxins are secondary metabolites produced by certain filamentous fungi, causing human and animal health issues upon the ingestion of contaminated food and feed. Among the safest approaches to the control of mycotoxigenic fungi and mycotoxin detoxification is the application of microbial biocontrol agents. *Burkholderia* *cepacia* is known for producing metabolites active against a broad number of pathogenic fungi. In this study, the antifungal potential of a Qatari strain of *Burkholderia cepacia* (QBC03) was explored. QBC03 exhibited antifungal activity against a wide range of mycotoxigenic, as well as phytopathogenic, fungal genera and species. The QBC03 culture supernatant significantly inhibited the growth of *Aspergillus carbonarius, Fusarium culmorum* and *Penicillium verrucosum* in PDA medium, as well as *A. carbonarius* and *P. verrucosum* biomass in PDB medium. The QBC03 culture supernatant was found to dramatically reduce the synthesis of ochratoxin A (OTA) by *A. carbonarius*, in addition to inducing mycelia malformation. The antifungal activity of QBC03’s culture extract was retained following thermal treatment at 100 °C for 30 min. The findings of the present study advocate that QBC03 is a suitable biocontrol agent against toxigenic fungi, due to the inhibitory activity of its thermostable metabolites.

## 1. Introduction

Mycotoxins are natural contaminants produced by certain filamentous fungi, mainly belonging to the genera *Aspergillus*, *Penicillium*, and *Fusarium*. The contamination of food commodities with toxigenic fungal species, either at pre- or post-harvest (transportation, storage) phases, leads to the accumulation of their toxic secondary metabolites [1,2]. Among a long list of mycotoxins, aflatoxins (AFs), ochratoxin A (OTA), deoxynivalenol (DON), trichothecene (T2) and 3′-Hydroxy T2 (HT2) citrinin, patulin, fumonisins, zearalenone, and trichothecenes are widely studied [3]. The health implications due to dietary exposure to mycotoxins include immunosuppression, carcinogenicity, mutagenicity, teratogenicity, genotoxicity, etc. [3,4,5,6].

OTA, the most toxic among the ochratoxins, is a secondary metabolite of some *Aspergillus* (*A. carbonarius*, *A. ochraceus*, *A. westerdijkiae* and *A. niger*) and *Penicillium* (*P. verrucosum*, *P. nordicum*) species [7]. The contamination of these fungi leads to the accumulation of mycotoxin in a variety of food and feed products including cereals, fruits juices, animal feed, wine, and baby food [8,9,10]. OTA is primarily known for its nephrotoxic activity, while the other effects range from the mild reduction in animal production performance to carcinogenesis [11]. In most developed, and in some developing countries, strict regulatory measures are in practice to monitor and control OTA in food and feed. The International Agency for Research on Cancer (IARC) has placed OTA in group 2B of the list of possible human carcinogens [12].

Apart from the toxigenic potential of the fungi, several other environmental factors—such as nutrient availability, humidity, temperature, and the pH of the substrate—play a vital role in the accumulation of toxins [13,14,15]. Several approaches, including the use of chemicals (pesticides and fungicides), physical interventions and improved managemental technologies are in practice to reduce/eliminate the fungal disease burden [16]. Each approach holds its success rate, but the side effects on the environment and production system have been compromised to some degree. It is known that the intensive use of chemicals not only raises human health concerns due to their residual transfer to food and feed, but it also leads to the emergence of fungicide-resistant populations. In recent years, research efforts were focused to find out safe approaches to overcome the problems related to mycotoxigenic fungi. Many microbial antifungal environment-friendly products (both volatiles and non-volatiles) are being explored and tested for their potential application in agriculture and the food industry [17,18,19,20]. The mode of action of these biological agents is either antibiosis (through the production of antibiotics, lytic enzymes, and antagonistic proteins), competition for space and nutrients, and/or the enhancement of plant defense mechanisms [21]. Several bacterial biocontrol agents including *Lactobacillus*, *Pseudomonas*, and *Bacillus* spp. have been explored for the ability to produce antifungal compounds that can lyse either the fungal cell wall or cell membrane [22].

In the present study, we aimed to explore the antifungal potential of *Burkholderia cepacia* strain QBC03, isolated from the Qatari feed market, against key mycotoxigenic and plant pathogenic fungi belonging to genera *Aspergillus*, *Penicillium* and *Fusarium*.

## 2. Results

### 2.1. Determination of the Spectrum of Burkholderia Cepacia QBC03 Antifungal Activity

**Figure 1:**
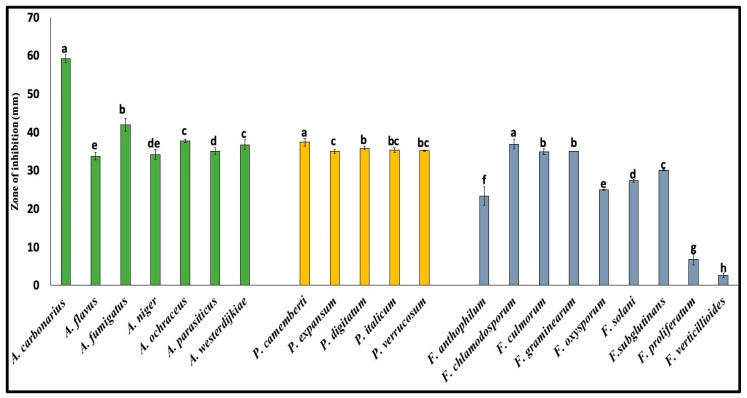
Effect of QBC03’s antifungal compounds on fungal growth. The zones of fungal growth inhibition (mm) around the bacterial colonies were recorded at day 4 of co-incubation. *A. carbonarius* showed highest sensitivity, while *F. verticillioides* was the least sensitive fungus. The Tukey test was performed to compare the inhibition zones for species within the same genus. Bars show the zone of inhibition (mm) with denoted letters (a–h). Values sharing the same letter are non-significantly different from each other (*p* ≤ 0.05).

The antagonistic spectrum of QBC03 was explored against 21 fungal species belonging to the *Aspergillus*, *Fusarium* and *Penicillium* genera, using a spore overlay method. The bacterial compound diffused into the medium showed a clear zone of fungal growth inhibition against a wide range of fungi, as shown in Figure 1. Among the tested fungal genera, *Aspergillus* and *Penicillium* showed higher sensitivity compared to *Fusarium*. Based on the zone of fungal growth inhibition, *A. carbonarius* was the most sensitive species. Similarly, *P. camemberti* showed significantly higher sensitivity as compared to other tested *Penicillium* spp. As can be seen from Figure 1, *F. verticillioides* was the least sensitive species.

### 2.2. Antifungal Activity of QBC03’s Culture Extract in Solid Media

**Figure 2:**
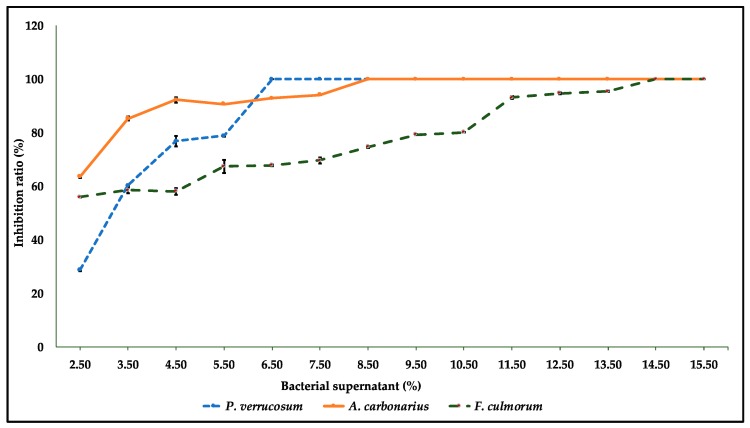
Comparison of the growth inhibition ratios for *A. carbonarius*, *F. culmorum* and *P. verrucosum* on PDA amended with the QBC03 culture supernatant. The PDA was amended with 2.5%, 3.5%, 4.5%, 5.5%, 6.5%, 7.5%, 8.5%, 9.5%, 9.5%, 10.5%, 11.5%, 12.5%, 13.5%, 14.5%, and 15.5% of the QBC03 culture supernatant, and the fungal colony diameter (mm) was measured after 5 days of incubation at 25 °C. The fungal colony size on treated media was compared with that on untreated PDA media to calculate the ratio of inhibtition (%).

To further explore the antagonistic activity of QBC03, a bacterial-cell-free culture extract was added to PDA at different concentrations, ranging from 2.5% to 15.5%. One fungal species from each tested genus was point-inoculated on solid media. A gradual inhibition of the fungal radial growth was noticed at increasing concentrations of the bacterial culture extract. There was complete inhibition of *P*. *verrucosum, A. carbonarius* and *F. culmorum* spore germination on PDA containing bacterial culture extract at concentrations higher than 5.5%, 7.5% and 13.5%, respectively (Figure 2). The PDA plates with QBC extracts above the threshold levels inhibited fungal spore germination, even after one month of incubation, hence indicating the long-term stability of the antagonistic compounds.

### 2.3. Effect of QBC03’s Metabolites on the Fungal Biomass

**Figure 3:**
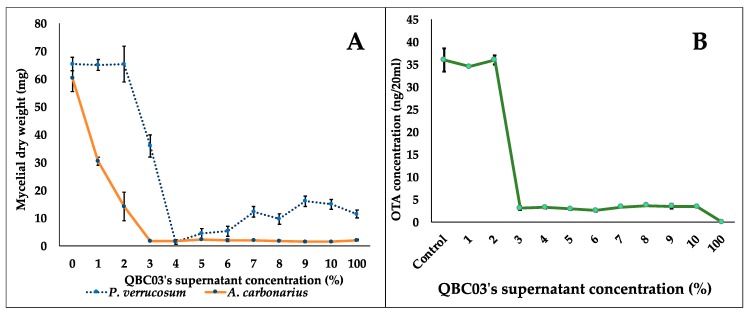
Effect of increasing QBC03 culture supernatant on fungal biomass and OTA synthesis in liquid media. Dry weight of the fungal mycelium (mg) in a medium inoculated with 0%, 1%, 2%, 3%, 4%, 5%, 6%, 7%, 8%, 9%, 10%, and 100% of QBC03 culture supernatant in PDB (**A**). OTA concentration (ng/20 mL PDB) synthesized by *A. carbonarius* in media containing the bacterial supernatant at levels given above (**B**).

To analyze the antifungal activity of QBC03 in a liquid medium, fungal spores of *A. carbonarius* and *P. verrucosum* were inoculated in PDB amended with increasing concentrations of the bacterial-cell-free culture supernatant. The fungal biomass, mycelial morphology and OTA synthesis (by *A. carbonarius* only) were recorded after 72 h of incubation with shaking (approximately 0.55 xg). The addition of 1% and 2% of bacterial extract showed a drastic effect on the biomasses of *A. carbonarius* and *P. verrucosum*, respectively, which decreased to half the biomass of the control. The biomass decrease displayed a dose-dependent manner (Figure 3A). Likewise, the effect of increasing bacterial concentrations was also found to affect the synthesis of OTA by *A. carbonarius*. As shown in Figure 3B, the concentration of OTA synthesized in the liquid media by *A. carbonarius* was directly related to its biomass. Following the fungal biomass pattern, a complete inhibition of OTA synthesis by *A. carbonarius* was noted in media containing 100% bacterial supernatant.

**Figure 4:**
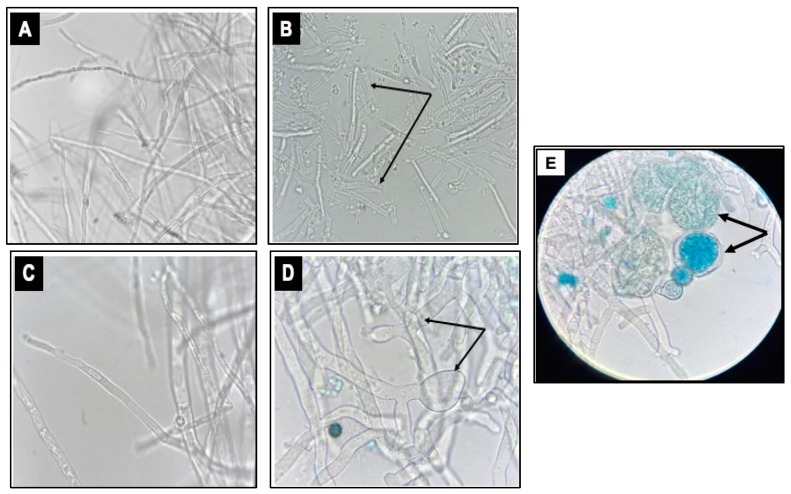
Effect of QBC03 culture supernatant on fungal mycelium morphology. Microscopic appearance of untreated *P. verrucosum* mycelium with intact long and thin cells (**A**), as compared to the fragmented mycelia ((**B**), arrows) of fungi treated with 2% of the bacterial extract. The morphology of *A. carbonarius* mycelia with long and thin cells from control (**C**) vs treated with 2% QBC03 extract ((**D**), arrows and (**E**), arrows). The images were captured at 1000× using a light microscope.

The microscopic examination of fungal mycelia, collected from the PDB media flask amended with increasing concentrations of QBC03 culture extract, showed significant morphological alterations. The mycelia of *P. verrucosum* obtained from PDB amended with 2% QBC03 extract (Figure 4B) showed the fragmentation and shortening of most of the fungal cells with thicker walls, as compared to the untreated mycelia (Figure 4A). Similarly, Figure 4D,E show that the addition of the QBC03 extract at 2% in PDB resulted in the thickening of mycelial walls with swollen ends and bulbous protoplasmic aggregations in *A. carbonarius*, compared to the thin long mycelia of the control fungi (Figure 4C). 

### 2.4. Effect of QBC03 Culture Extract on Fungal Spore Germination

**Figure 5:**
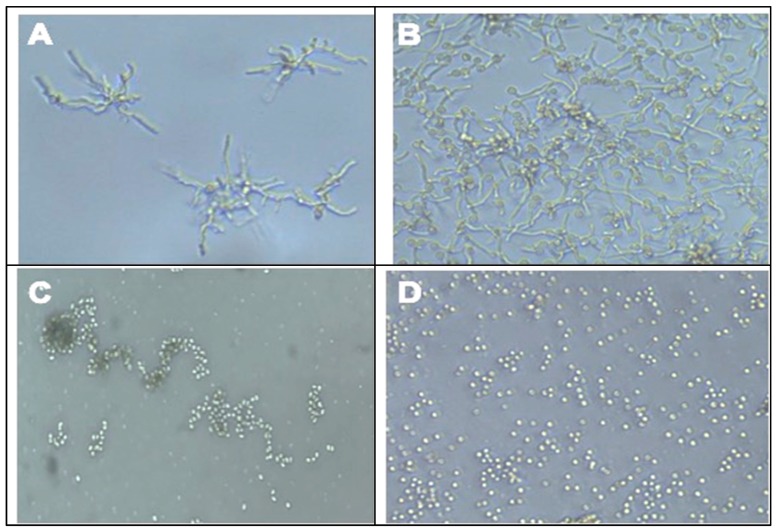
Effect of QBC03’s supernatant on the conidiospore germination. *P. verrucosum* control spores showing germination tubes (**A**), as compared to those exposed to bacterial antifungal compounds (**C**) showing no germination after 24 hr of incubation. Plate (**B**,**D**) are *A. westerdijikae* spores in PDB with visible spore germination, and in PDB+QBC03 culture supernatant without any germination, respectively. The images were captured using an inverted microscope (×600).

The effect of QBC03 metabolites on the spore germination of the selected toxigenic fungal species—*P. verrucosum*, *A. westerdijikae*, *A. carbonarius* and *F. oxysporum*—was explored. In the wells of a 24-well plate, fungal spores were suspended in PDB mixed with the bacterial culture supernatant. The conidial germination was observed under a microscope and the representative images are presented in Figure 4. After 24 h of incubation, the conidia of the control groups (Figure 5A,B) showed long, protruding germination tubes, which were not found in the treated conidia (Figure 5C,D).

### 2.5. Study on the Thermostability of QBC03 Antifungal Compounds

**Figure 6:**
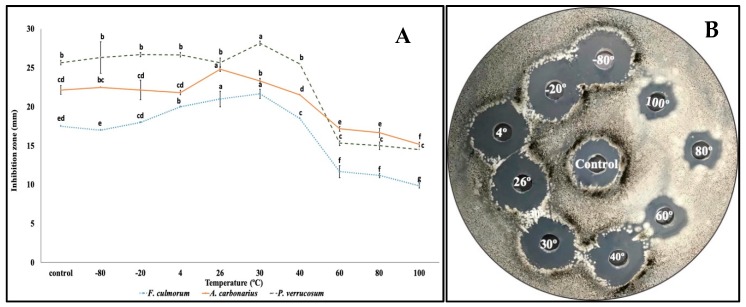
Effect of temperature on the stability of the QBC03 supernatant. The zones of fungal growth inhibition (mm) against *F. culmorum*, *A. carbonarius* and *P. verrucosum* were recorded around the wells containing heat-treated bacterial culture extract. (**A**) Inhibition zones at different temperature treatments for each species were denoted with letters (a–g), and inhibition zones sharing the same letter within the same species are considered as insignificantly different from each other. (**B**) Representative Petri dish of *A. carbonarius* showing the inhibition zones.

The thermostability of QBC03 antifungal compounds was studied by exposing its cell-free culture supernatant to different temperatures. Low-temperature treatments, such as −80 °C, −20 °C, and 4 °C for 30 min, had no significant effect on the antifungal activity of the bacterial supernatant (Figure 6A). The QBC03 culture supernatant, treated at 26 °C for 30 min, showed the highest activity against *A. carbonarius* (Figure 6A,B), and the one treated at 30 °C showed the most prominent activity towards *P. verrucosum* and *F. culmorum*. Increasing the heat treatments, at 40 °C and above, showed a decline in the antifungal efficacy of the bacterial compounds. Thermal treatment at 100 °C for 30 min, although significantly reducing the antifungal activity of the bacterial culture extract, still resulted in significant fungal growth inhibition.

## 3. Discussion

In this study, we aimed to explore the antifungal potential of a *B. cepacia* strain (QBC03) isolatedfrom Qatar against some key mycotoxigenic and plant pathogenic fungi. In preliminary screening experiments, the antagonistic activity of QBC03 was tested on a total of 21 fungal species belonging to the genera *Aspergillus, Fusarium* and *Penicillium*. Although QBC03 showed a strong antagonistic activity against all the tested fungal species, some genus- and species-specific differences were observed. *Aspergillus* and *Penicillium* spp. were more sensitive in comparison to *Fusarium* spp. Similarly, within the same genus, some species were more sensitive than others, which might be due to structural or chemical differences in the cells. In line with the present study, the volatiles produced by *Bacillus megaterium* and *Pseudomonas protegens* showed differential effects on *Aspergillus* and *Penicillium* spp. [23].

In order to further explore the nature of the antifungal compounds, the culture supernatant of QBC03 was added at increasing concentrations to a PDA medium. Increased concentrations of the bacterial culture supernatant in the medium resulted in the increased growth inhibition of the tested fungi. There was complete inhibition of *P. verrucosum, A. carbonarius* and *F. culmorum* on the PDA medium amended with ≥6.5%, ≥8.5% and ≥14.5% of QBC03 culture extract, respectively. These results suggest the non-volatile nature of the antagonistic compounds released by the bacteria. In line with the present study, Kilani-Feki et al., [24] reported a 90% inhibition ratio of *Rhizoctonia solani* in PDA amended with 3% culture supernatant of *B. cepacia* (CS5). Similarly, the culture extract of *Bacillus subtilis* showed similar antagonistic activity against plant pathogenic *Stenocarpella maydis* and *Stenocarpella macrospora* [25].

In another experiment, we aimed to explore the effect of the bacterial culture supernatant on fungal biomass, mycelium morphology and mycotoxin biosynthesis. The incorporation of very low percentages (1% and 3%) of bacterial supernatant reduced the biomass significantly to almost half of that observed in the untreated control of both *A. carbonarius* and *P. verrucosum*. A similar effect was observed on *Botrytis cinerea* biomass production upon the addition of a *B. cepacia* supernatant at 0.9% [26]. Accordingly, with regards to biomass reduction, a similar decline was observed in OTA content released in the culture medium, indicating the significant effect of QBC03 metabolites on fungal growth as well as their mycotoxin production. These effects may be associated with the downregulation of fungal genes responsible for growth and mycotoxin synthesis potential. In a recent study [27], the exposure to volatile yeast (2-phenylethanol) resulted in a significant reduction (25%–99.9%) in the expression of key genes involved in OTA synthesis.

Future studies will be designed to investigate the precise nature of QBC03 antifungal compounds and their mode of action. The microscopic observation of the fungal mycelium exposed to QBC03 culture supernatant showed fragmentation, granulations and swellings. In addition, in the present study, the appearance of chlamydospores (Figure 4E), which are a general indication of fungal growth suspension and dormancy [28,29], showed that the QBC03 culture supernatant created an environment not favorable for the growth and propagation of *A. carbonarius*.

The absence of a germination tube emerging from the surface of conidia indicated that bacterial compounds completely inhibited the spore germination in *P. verrucosum, A. westerdijikae* and *F. oxysporum*. These findings are in line with earlier studies [30,31], reporting similar spore germination inhibition by bacterial antifungal compounds. The ability of the QBC03 antagonistic compounds to maintain their antifungal activity, even after boiling for 30 min, is the most promising feature for their possible application in future. The thermostability of bacterial compounds towards their antagonism against fungal isolates has been studied [32,33,34,35].

## 4. Conclusions

In this study, the in-vitro biocontrol activity of a *B. cepacia* strain (QBC03) against a wide range of fungi belonging to the genera *Aspergillus, Penicillium* and *Fusarium* was investigated. A high sensitivity to QBC03 was observed in fungi belonging to the *Aspergillus* genus, followed by *Penicillium* and *Fusarium*. The presence of bacterial antifungal compounds in the culture supernatant confirmed the diffusible nature of molecules. The thermostability of the QBC03 compound under a wide range of temperatures warrants its wide application. The promising antagonistic activity of QBC03 against a range of fungi suggests its application in the agriculture and the food industry, to replace or to strongly reduce the application of chemical fungicides. We believe that the findings of this research represent an excellent basis for the future exploitation of these antifungal compounds, hence, more studies need to be focused on purifying these interesting antifungal compounds and investigating their nature.

## 5. Materials and Methods

### 5.1. Microbial Strains

The *Burkholderia cepacia* strain (QBC03) is a local strain isolated from marketed feed samples in Qatar (data not shown). The list of strains included; *A. carbonarius* (AC82), *A. flavus* (CECT2687), *A. fumigatus* (AF14), *A. niger* (AN8), *A. ochraceus* (CECT2948), *A. parasiticus* (AF82), *A. westerdijkiae* (AW82), *P. camemberti* (PC44), *P. expansum* (PE82), *P. digitatum* (PD43), *P. italicum* (PI48), *P. verrucous* (TF11), *F. anthophilum* (FAn01), *F. chlamydosporum* (FCh01), *F. culmorum* (FCu11), *F. graminearum* (FGr14), *F. oxysporum* (Fox9), *F. solani* (FS05), *F. subglutinans* (FSuF12), *F. proliferatum* (FP08), and *F. verticillioides* (FV04).

### 5.2. Screening the Antifungal Activity of QBC03

The antifungal spectrum of QBC03 was determined using the spore overlay method around the bacterial colony. Briefly, with the help of a sterile toothpick, bacterial cells were transferred to the center of a nutrient agar plate, prepared by adding 1 g of meat extract (Mikrobiologie, Darmstadt, Germany), 5 g of peptone (Acumedia, Heywood, UK), 5 g of sodium chloride, 2 g of yeast extract (Himedia, Mumbai, India) and 15 g of agar in 1L of water. Before overlying the fungal spores, bacterial plates were incubated at 30 °C for 48 h to allow sufficient time to synthesize their antagonistic compounds. From a 7 d old, pure fungal colony, a loopful of inoculum was taken and suspended in 0.9% NaCl with 1% Tween 80. The spores were counted using a hemocytometer and their concentration was adjusted to 10^6^/mL. The spores were transferred to 10 mL of soft PDA (Potato Dextrose agar from Foremedium, Hunstanton, England) and assayed around the bacterial colony. The plates were incubated at 26 °C for four days, and the diameters of the fungal zones of inhibition around the bacterial colony were measured in mm. The antifungal spectrum of QBC03 was tested against single representatives of the 21 fungal species mentioned in Section 5.1.

### 5.3. Investigation of QBC03 Culture Extract on the Fungal Growth in Solid Media

To test the antifungal activity of the QBC03 culture supernatant, bacterial cells were cultured in Nutrient Broth Yeast (NBY) extract media as described by Kilani-Feki and Jaoua [17]. For this purpose, a 7 h old preculture of QBC03 was prepared by transferring a single colony to 10 mL NBY broth, and from this an inoculum was taken to prepare a 48 h-old bacterial culture in NBY. The bacterial broth was centrifuged at 5500× g for 20 min and the supernatant was collected. The obtained supernatant was added to molten PDA at 2.5%, 3.5%, 4.5%, 5.5%, 6.5%, 7.5%, 8.5%, 9.5%, 9.5%, 10.5%, 11.5%, 12.5%, 13.5%, 14.5%, and 15.5%. To inhibit the growth of any bacterial cell in the media, chloramphenicol at 100 μg/L was also added. From each fungal genus, one species (*A. carbonarius*, *F. culmorum*, *P. verrucosum*) was chosen for this experiment. Three microliters of fungal spore suspension (×10^3^) was inoculated on the center of the PDA plates which were subsequently incubated at 26 °C for 5 days. The diameter of the fungal colonies was measured at day 5, and the inhibition ratios were estimated using the following equation:

$$\mathrm{Inhibition}\;\mathrm{ratio}\;\left(\%\right)=\frac{\mathrm{diamter}\;\mathrm{of}\;\mathrm{control}-\mathrm{diameter}\;\mathrm{of}\;\mathrm{treated}}{\mathrm{diameterof}\;\mathrm{the}\;\mathrm{control}}\times 100$$

### 5.4. Investigation of the Effect of QBC03 Culture Extract on Fungal Biomass and Mycotoxin Synthesis

The effect of the bacterial culture supernatant on the fungal biomass production and OTA synthesis of *A. carbonarius* was studied. The bacterial culture supernatant was obtained as described in Section 5.3, and was added to PDB (Potato Dextrose Broth from Foremedium Hunstanton, England) amended with 500 μg/L chloramphenicol to obtain the final concentrations of 0%, 1%, 2%, 3%, 4%, 5%, 6%, 7%, 8%, 9%, 10%, and 100% (no PDB, only bacterial culture supernatant with antibiotic). Inocula of *A. carbonarius* or *P. verrucosum*, represented by 10 μL of a fungal spore suspension (10^6^/mL), were transferred to flasks (50 mL volume) and incubated at 26 °C for 72 h with continuous shaking at approximately 0.55× *g*. The fungal biomass production was measured by the filtration of flask contents using nitrocellulose paper in a Buchner funnel connected to a vacuum assembly. The biomass of the treated fungi was compared with the untreated control (only PDB). Morphological changes in the fungal mycelium including hyphal cell fragmentation, cell wall thickness, cytoplasmic degranulation, and the formation of chlamydospores were observed using a light microscope. The filtrate of each treatment was collected separately in Eppendorf tubes for the analysis of mycotoxin concentration using an OTA ELISA kit (RIDASCREEN^®^ Ochratoxin A, R-Biopharm, Germany), and the readings were taken using an ELISA plate reader installed with SkanIt software.

### 5.5. Evaluation of the Effect of QBC03’s Antifungal Compounds on Fungal Spore Germination

The effect of the QBC03 extract on fungal spore germination was tested in 24-wells plates. Spore suspensions were prepared from 7 day-old cultures and washed twice [22]. PDB (900 μL), amended with 500 μg/L chloramphenicol and QBC03 culture supernatant (100 μL), was added in each well. Further, 2 μL of fungal spores (×10^3^) from either *P. verrucosum, A. westerdijikae* or *F. oxysporum* were added to each well. The plate was sealed with Parafilm and incubated at 26 °C for 24 h. The germination of the spores treated with the extract was compared to the germination of the spores in control (having PDB only).

### 5.6. Influence of Temperature on the Stability of QBC03 Antifungal Compounds

In order to test the thermal stability of the QBC03 antifungal compounds, the bacterial culture supernatant—obtained using the method described in Section 5.2—was treated at −80 °C, −20 °C, 4 °C, 26 °C, 30 °C, 40 °C, 60 °C, 80 °C, and 100 °C for 30 min. Spore suspensions from *A. carbonarius, P. verrucosum* and *F. culmorum* were prepared (10^6^/mL), and 200 μL was spread on the surface of PDA amended with 100 μg/L chloramphenicol. Wells of 7 mm in diameter were obtained using a sterile cork-borer in the PDA plates, and 100 µL of treated extract was loaded. After 72 h, the zones of fungal growth inhibition around the wells were measured.

### 5.7. Statistical Analysis

SPSS statistical software (Version 23, IBM, NY, USA, 2017) was used for data analysis. An analysis of variance (ANOVA) and the multiple comparisons test (Tukey-test) were performed.
